# “My greatest fear is that he’ll get diabetes”: a qualitative study of families’ psychosocial responses to early detection of type 1 diabetes

**DOI:** 10.3389/fcdhc.2026.1796164

**Published:** 2026-06-12

**Authors:** Pernille Bech Flarup, Dan Grabowski, Patricia DeCosta, Julie Christine Antvorskov, Flemming Pociot, Louise Norman Jespersen

**Affiliations:** 1 Department of Prevention, Health Promotion & Community Care, Copenhagen University Hospital – Steno Diabetes Center Copenhagen, Herlev, Denmark; 2 Department of Public Health, University of Southern Denmark, Odense, Denmark; 3 Translational Type 1 Diabetes Research, Department of Clinical Research, Steno Diabetes Center Copenhagen, Herlev, Denmark; 4 Department of Clinical Medicine, Faculty of Health and Medical Science, University of Copenhagen, Copenhagen, Denmark

**Keywords:** children, early detection, psychosocial aspects, sense of coherence, type 1 diabetes, first-degree relatives, qualitative research

## Abstract

**Background:**

The incidence of type 1 diabetes (T1D) among European children has nearly doubled over the past three decades. Emerging disease-modifying therapies highlight the importance of early detection to delay disease onset and enhance psychosocial preparedness. However, participation in early detection may also evoke parental anxiety, underscoring the need to understand families’ psychosocial experiences to ensure ethically and emotionally responsive screening programs. This study explores how families experience participation in early detection when children are first-degree relatives of individuals with T1D.

**Methods:**

Semi-structured interviews were conducted with 31 parents of children aged 1–17 years participating in a Danish early detection program for T1D (DiaUnion). Data were analyzed using Antonovsky’s Sense of Coherence framework to examine how families perceive, manage, and make sense of disease-related uncertainty during participation.

**Results:**

Three themes describe how families experience participation in early detection. First, The Family Narrative illustrates how parents’ prior experiences with T1D shape expectations and emotional responses to screening, often combining practical knowledge with heightened concern. Second, Diabetes in Everyday Life shows how families integrate early detection into daily routines and draw on experiential knowledge to manage uncertainty and interpret potential symptoms. Third, Children’s Involvement highlights how parents navigate decisions about how and when to involve their children in discussions about risk and screening. Across themes, clear communication and procedural transparency were central in supporting families’ sense of coherence and their ability to manage uncertainty.

**Conclusion:**

Early detection programs for T1D should attend not only to clinical accuracy but also to families’ psychosocial needs. Clear communication, transparency, and structured psychosocial support are essential to strengthen families’ capacity to manage uncertainty and ensure that early detection is both effective and acceptable.

## 1 Introduction

Type 1 diabetes (T1D) remains a growing global health challenge, with an estimated 9.5 million people affected worldwide in 2025 and projections suggesting this number will rise to 14.7 million by 2040 (1). In Europe, the trend is particularly noteworthy: Among children aged 0–14 years, incidence rates have nearly doubled, increasing from 10.85 per 100,000 person-years between 1994 and 2003 to 20.96 per 100,000 between 2013 and 2022. This upward trajectory is evident across most European countries, with the most pronounced increases observed in Northern Europe (2).

Recent breakthroughs, including the development of disease modifying immunotherapies, have demonstrated that the clinical onset of type 1 diabetes (T1D) can be delayed by several years (3). These advances have intensified calls for comprehensive screening programs that allow for earlier identification of individuals at risk and create opportunities for timely intervention aimed at preserving residual beta cell function. Such strategies hold the potential to reduce the risk of diabetic ketoacidosis (DKA) at diagnosis and to improve long term outcomes, including lower lifetime HbA1c levels and a reduced risk of complications (4–6). At the time this study was conducted, however, no preventive or disease modifying treatment to delay T1D onset had been approved for use in Europe, and screening initiatives therefore primarily served prognostic and preparatory purposes rather than offering immediate therapeutic benefit. Following completion of data collection (August 2025), this context shifted, as the European Commission granted EU-wide marketing authorisation for teplizumab (Teizeild; Tzield out-side the EU) in January 2026, marking the first approved intervention to delay progression to stage 3 type 1 diabetes in individuals with stage 2 disease (7).

Reflecting this growing momentum, the International Society for Pediatric and Adolescent Diabetes (ISPAD) recommends integrating screening programs into healthcare systems as part of standard care (8). These guidelines aim not only to delay or prevent the clinical onset of T1D, but also to prepare families for a smoother transition to insulin therapy. Screening is thereby no longer concerned solely with identifying disease earlier, but addition-ally with acknowledging the psychosocial realities families face and ensuring they are emotionally supported and equipped to navigate the challenges of a chronic condition (8).

The emphasis on psychosocial preparedness is particularly salient given previous evidence showing how parents often feel shocked, overwhelmed, and unprepared at the time of their child’s diagnosis—especially when it occurs in the context of diabetic ketoacidosis (DKA), which is associated with heightened psychological distress and impaired disease management (9, 10). International studies, however, have shown that participation in screening programs itself can provoke parental anxiety. The trend is particularly evident among families with children who have tested positive for autoantibodies (11, 12). Johnson et al. (12) further demonstrated that, while this anxiety tends to diminish over time, it can persist in families whose children present with multiple autoantibodies—underscoring the need for targeted psychosocial support for high-risk groups. This heightened anxiety may be further intensified by the unpredictability of disease onset and the fact that, until now, early detection has largely occurred in the absence of concrete preventive measures. For parents of children at risk of serious illness, uncertainty is widely recognized as a central contributor to stress (13).

Longitudinal data, at the same time, suggest that children identified as being at risk for T1D who have participated in research involving monitoring, exhibit improved diabetes-related quality of life, and that their parents experience lower stress post-diagnosis compared to those diagnosed in the broad population. This may reflect the fact that families engaged in follow-up studies, despite some uncertainty during the waiting period, are better prepared for a potential diagnosis (14). Sociodemographic factors, as highlighted by Melin et al. (15), add an-other layer to understanding family experiences in early detection contexts, as parental anxiety levels were assessed five years after screening and showed that parents’ educational level influences their level of concern.

Considering the emerging era of early detection, further knowledge in this area is essential to developing family interventions that not only reduce parents’ anxiety but also promote resilience and improve preparedness for a potential diagnosis. The present study aims to further examine the emotional and psychological dimensions associated with identifying T1D risk among first-degree relatives.

## 2 Aim

The purpose of our study was to explore how families perceive and experience participation in an early detection program for T1D. Using a qualitative approach, the study focuses on the emotional, psychological, and practical challenges associated with early detection, as well as areas where families feel most vulnerable and in need of healthcare support. By focusing on parents’ lived experiences, the study aims to contribute knowledge to guide the development of early detection programs that are not only clinically sound but also ethically and emotionally attuned to families’ needs.

## 3 Method

### 3.1 Setting

EDENT1FI (European action for the Diagnosis of Early Non-clinical Type 1 diabetes For disease Interception) is a pan-European initiative aimed at identifying children in the early, presymptomatic stages of T1D through large-scale early detection of diabetes-associated auto antibodies (16). Early detection programs are currently being implemented through the EDENT1FI initiative in 13 countries, including sites in Austria, Belgium, Czechia, Denmark, Finland, France, Germany, Italy, the Netherlands, Poland, Portugal, the UK, and the US. Between November 2023 and October 2028, approximately 200,000 children and adolescents aged 1–17 years are expected to participate (Edent1fi.eu) (17).

Within this European framework, DiaUnion represents the Swedish/Danish collaboration be-tween Steno Diabetes Center Copenhagen, Lund University, and Medicon Valley Alliance, aiming to offer early detection of T1D (Edent1fi.eu) (17). The present study was conducted in as-sociation with DiaUnion Denmark, where the early detection process is limited to first-degree relatives of individuals with T1D. In contrast, Sweden offers population-based early detection that includes children regardless of family history (18).

In Diaunion, children are tested for diabetes related islet autoantibodies, which are validated markers of autoimmune activity preceding clinical T1D. A negative result indicates a low current risk but does not rule out future development of T1D, as autoantibody status may change over time (19). Children with a first degree relative with T1D have a markedly higher risk of developing the disease, with a lifetime risk of approximately 5%, compared with about 0.3–0.4% in the general population, corresponding to a 10–15 fold increased risk (19). A positive result indicates the presence of one or more islet autoantibodies and is associated with an increased risk of developing type 1 diabetes (T1D). Risk estimates depend on the number of autoantibodies detected: the presence of a single autoantibody is associated with an approximately 15% risk of progression to clinical disease within 10 years. In contrast, the presence of multiple autoantibodies is associated with a substantially higher risk—around 44% within 5 years—and a lifetime risk approaching 100% (4, 19, 20). Identification of autoantibody positive children enables close monitoring and may reduce the risk of severe metabolic complications as well as long term complications associated with T1D (21). In addition, participants in DiaUnion are also tested for markers related to celiac disease and autoimmune thyroid disorders (22).

### 3.2 Design

The study employed a qualitative design using semi-structured interviews to explore parents’ experiences and reactions related to the early detection process. The interview guide included 16 questions plus follow-up prompts, which covered the following broad themes:

-Family Introduction.

-Motivation & Experience of the Process.

-Preparation, Information & Risk Perception.

-Emotions, Thoughts & Concerns.

-Family Dynamics & Children’s Reactions.

-Results & Follow-up.

-General Reflections & Considerations.

### 3.3 Recruitment procedure

The study cohort included two distinct participant groups from DiaUnion: parents of children aged 1–17 years who had undergone the early detection process, and adults aged 18–42 years who themselves had undergone the early detection process. The present paper focuses exclusively on the data from interviews with parents of children aged 1–17 years.

The participants were randomly selected in Redcap based on the timing of the blood sample, the aim being to include families and adults at different stages of the process. Recruitment involved phone calls, voicemail messages, and text messages containing information about the study and the option to respond via the same channels.

#### 3.3.1 Participant overview


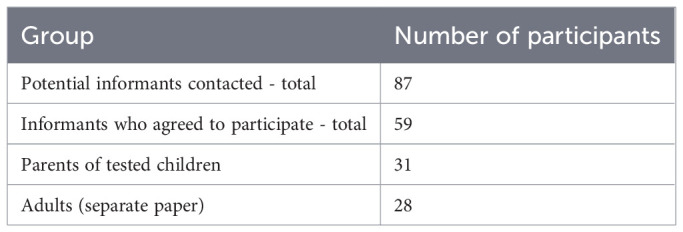


The parents who participated in our study expressed that their primary motivation stemmed from a personal concern for their children’s health. However, many also emphasized a strong desire to contribute to scientific research. Their existing relationships with Steno Diabetes Center played a significant role in fostering trust and a sense of reciprocity, which further encouraged their involvement. This collective engagement is reflected in the study’s participation rate: Of the 87 individuals invited, 59 agreed to participate, corresponding to a participation rate of approximately 67.8%. This reciprocal relationship between first-degree relatives and diabetes research reveals an important dynamic that could inform future strategies for implementing early detection initiatives outside of high-risk groups.

### 3.4 Data collection

Participants were purposively sampled based on their stage in the early detection process, as recorded in the REDCap database. Efforts were made to ensure a balanced distribution between parents who were awaiting test results and those who had already received them. Fifteen interviews involved parents still awaiting results, while sixteen involved parents who had received them. Among these sixteen families, seven had received a negative result, whereas nine had received a result indicating that the child tested positive for one or more autoantibodies. Families with positive results were deliberately oversampled to gain deeper insight into the psychosocial aspects of prolonged uncertainty and follow-up.

Data collection took place between May 1 and August 15, 2025. Semi-structured interviews were conducted either via telephone or in person, through home visits or meetings at Steno Diabetes Center Copenhagen by author PBF. In total, 23 telephone interviews and 8 in-person interviews were conducted. Interviews lasted between 21 and 53 minutes, were audio-recorded, and transcribed verbatim by student research assistant EBP.

Telephone interviews were primarily chosen for pragmatic reasons, including the geographic dispersion of participating families and the aim to minimize participant burden. This format allowed greater flexibility in scheduling and enabled parents to participate from their own homes, which was also considered relevant given the potentially sensitive nature of the study topic. Face-to-face interviews were conducted when feasible and based on participant preference.

No systematic differences were observed between telephone and in-person interviews regarding interview length, depth of reflection, or the themes identified. Participants in both formats appeared equally able to articulate their experiences. While in-person interviews allowed for observation of nonverbal cues, telephone interviews may, in some cases, have facilitated openness by providing a greater sense of privacy and anonymity. Both formats were therefore considered methodologically appropriate for the study aims.

### 3.5 Measures

Demographics and characteristics of the participating parents are presented in **Table 1**:

**Table 1:** Characteristics of included parents.

Variable	Frequency (%)
Parent gender and T1D status	
Mothers (total)	20 (64.5%)
└ with T1D	13 (41.9%)
└ without T1D	7 (22.6%)
Fathers (total)	11 (35.5%)
└ with T1D	8 (25.8%)
└ without T1D	3 (9.7%)
Educational level	
Long-cycle higher education	16 (51.6%)
Medium-cycle higher education	8 (25.8%)
Vocational/upper secondary	3 (9.7%)
Primary and lower secondary	2 (6.5%)
Unknown	2 (6.5%)
Marital status	
Married	22 (71.0%)
Divorced	2 (6.5%)
Cohabiting	4 (12.9%)
Unknown	3 (9.7%)
Place of residence	
Urban area	20 (64.5%)
Suburban area	9 (29.0%)
Rural area	2 (6.5%)

The families’ ethnic backgrounds were predominantly Caucasian. Three families reported having Indian, British, and Icelandic origins, respectively.

### 3.6 Ethics

The study was assessed by the Ethics Committee of the Capital Region of Denmark (journal number F-25026891) and approved by the Danish Regional Data Protection Agency. The study was conducted in accordance with the ethical principles of the Declaration of Helsinki (23).

During this initial contact, parents were informed about the purpose of the study, what participation would entail, and that participation was entirely voluntary. It was explicitly emphasized that declining participation or withdrawing from the study would have no consequences for the family’s or the child’s care. Parents who expressed interest in participating subsequently received written information about the study along with a written informed consent form. Written informed consent was obtained from all participants during REDCap prior to conducting the interviews.

Ethical considerations are particularly salient in early detection programs for T1D involving children, as participation may introduce uncertainty and vulnerability for families, especially given that no preventive treatment had been approved by the European Medicines Agency (EMA) at the time. To address these ethical challenges, particular attention was paid to participants’ emotional well-being throughout the study. Families—especially those receiving a positive screening result—were informed about available support from healthcare professionals before, during, and after participation. Interviews were conducted with sensitivity to participants’ emotional responses and with respect for the vulnerability inherent in navigating early detection in the absence of approved preventive treatment options.

### 3.7 Analytical approach

The interview data were analyzed using an inductive thematic analysis, as described by Braun and Clarke (24). In line with this approach, analysis was not limited to descriptive coding but involved an interpretative engagement with the data. Consequently, the results section presents themes that combine empirical excerpts with analytical interpretation. This approach reflects the understanding that themes in qualitative research are not simply discovered but actively constructed through the researchers’ analytic engagement with participants’ accounts. For transparency, illustrative quotations are provided throughout the results section to anchor interpretations in the empirical material.

Each phase of the analysis was discussed among the authors to ensure that consensus was reached regarding the main themes and subthemes. In Phase 1, authors LNJ and PBF jointly listened to and re-read the transcripts to gain an overall impression of the data. In Phase 2, authors LNJ and PBF were equally involved in the initial coding process, which was conducted by PBF using the qualitative data analysis software NVivo to organize the data into initial codes. In Phase 3, the authors PBF and DG explored the relationship between the codes and sorted them into potential themes and subthemes. In Phase 4, the authors PBF and DG evaluated and re-fined the themes, scrutinizing the data underlying them to ascertain whether they exhibited a consistent pattern. This involved iterative refinement of data excerpts to ensure coherence within each theme. In Phase 5, final main themes and subthemes were defined and named by PBF and DG. The analysis was informed by the Sense of Coherence (SOC) framework; however, coding was conducted inductively and remained fully data driven.

## 4 Theoretical framework

The theoretical framework underpinning the analysis is Sense of Coherence (SOC), a sociological framework concerned with how individuals manage stress and sustain well-being. SOC comprises three core components:

Comprehensibility – The ability to understand and make sense of life events.

Manageability – The belief that one has the resources to cope with challenges.

Meaningfulness – A sense that life has purpose, making difficulties worth overcoming.

Individuals with a strong SOC are better equipped to handle stress and adapt to adversity, leading to improved mental and physical health (25).

SOC was adopted during the analytic process, as parents’ narratives consistently emphasized efforts to make sense of screening results, manage uncertainty about future disease risk, and integrate this knowledge meaningfully into everyday family life. Although alternative qualitative frameworks address perceptions and management of health risks, SOC was considered particularly suitable as it captures how families actively create coherence and mobilize resources in situations characterized by uncertainty rather than manifest illness. In this study, SOC serves to illuminate how parents navigate the early detection process for type 1 diabetes in different ways, and how focused attention to key psychosocial aspects may help families feel supported and secure throughout and beyond the early detection process.

## 5 Findings

In the presentation of the results, each quote from parents will include information about two aspects:

1. Test status of the child: Waiting result = “W” or Answer received = “A”.
2. Diabetes status of the parent: Has diabetes = “+ diabetes” or No diabetes “- diabetes”.

This approach is intended to create transparency, allowing readers to clearly see whether the family is still awaiting the test result or has already received an answer, as well as whether the quote comes from a parent with or without diabetes. The code is placed immediately after the quote.

### 5.1 Theme 1: The Family Narrative

One main finding concerns how children’s symptoms and behaviors are interpreted through the lens of the parent’s own experience with T1D. Notably, the parents’ personal stories about diabetes emerge spontaneously when they are asked about their motivation for participating in early detection:

*“(…) I’ve always said that [child 1] is just like me—she started off with eczema when she was little, just like I did. Milk allergy, same as me—and you know, all those autoimmune reactions, which eczema also is. So, she was really the one I was most anxious about, so to speak (…)*.

*And yes, I know gut feelings aren’t always accurate—not at all, and I really hope mine aren’t. But it’s also more about prevention, you know? I walked around for quite a while, I was seriously ill, before it got really bad. So just being monitored—whether that long-term blood sugar is increasing, or whether something’s happening if the antibodies are there. I think it’s a huge advantage for my children in terms of complications. I couldn’t see out of my right eye at the end, and I was limping. I was really sick. I eventually collapsed on a lawn, and I lost 15 kilos in two months—it was crazy.”* (Mom1 +diabetes, W).

Mom1´s reflections reveal how her personal history with autoimmune conditions—eczema, milk allergy, and ultimately T1D—shapes her vigilance and concerns for her child’s health. Her daughter’s early symptoms are not viewed in isolation but are mapped onto a familiar trajectory, reinforcing a sense of anticipated risk. Despite acknowledging the fallibility of gut feelings, the mother’s intuition remains a guiding force, which offers her a framework for understanding and navigating uncertainty, particularly in the context of early detection. Her ac-count underscores the emotional and physical toll of delayed diagnosis, which in turn justifies her proactive stance on early detection. Mom1´s narrative seems to demonstrate how some parents strive to make potential health threats comprehensible and understandable by drawing parallels to their own lived experiences. Even when intuition is questioned, it might serve as a stabilizing mechanism—helping the parent interpret ambiguous symptoms and maintain a sense of control in an otherwise unpredictable situation.

It was evident in several of the interviews that behind the desire for clarity lay a strong wish among the parents to protect their child from illness—or at least, to feel more in control. This part of the process also echoed the parents’ own diabetes narratives:

*“Ever since we decided whether to have children at all, it’s been part of our considerations—because I really don’t think having diabetes is an easy thing, even though I’ve actually had my own insulin production for a very long time, and I think that puts me in the relatively “easy category.” Maybe I’ve had a slight professional advantage, but even so, it’s not something I’d wish on my worst enemy (…)”*.

*And I think it’s triggered a lot of worry in me, because I had a very slow onset of diabetes, I believe—and as far as I understand it, I also tested positive for that slow-progressing autoantibody. It was a very calm and gradual transition. I feel like it completely mirrors my own experience, but really, it’s my emotions that are triggered. It’s more a personal reaction than one based purely on professional insight.”* (Mom2 +diabetes, A).

Mom2 works within the healthcare sector and therefore brings professional knowledge of T1D and disease trajectories into her reflections. However, she explicitly distinguishes this professional insight from her emotional response. Although her own disease course has been relatively mild and gradual, and although her professional background provides her with medical understanding of early disease processes, she emphasizes that her worries are primarily rooted in her personal experience of being diagnosed with T1D. She explains that participation in the early detection program reactivates memories and emotions associated with her own disease onset, which she now perceives as mirrored in her children. Thus, while her professional background frames her understanding of the situation, her reactions are driven mainly by emotionally embodied experiences rather than by clinical assessment alone.

The kind of reflection offered by Mom2 can be understood through the concepts of comprehensibility and meaningfulness, as she attempts to make sense of the situation by interpreting symptoms and reactions through her own experiences and personal history with the ill-ness. SOC distinguishes between focused and diffuse emotions. Focused emotions—such as worry, grief, and guilt—are more compatible with perceiving a situation as comprehensible and are therefore more likely to activate coping mechanisms. In contrast, diffuse emotions—such as anxiety, rage, and despair—tend to trigger subconscious defense mechanisms and hinder coping. In this case, the mother demonstrates a high level of emotional awareness; she can identify and articulate her worries without feeling directly threatened by them. This suggests that her emotional responses serve as a resource in her coping process and contribute to a meaningful understanding of the early detection experience.

In both examples above, entering the early detection process for T1D appears to be a concrete strategy aimed at gaining greater control over an unpredictable situation concerning the child’s risk and development of the disease. This was a recurring tendency among the parents. Here a statement from a mother who had just received a negative test result for both of her children:

*“Well, it definitely means that we can relax for now—that I don’t have to, especially when he’s really sick, look at him and think, oh no, I just know you’re about to get diabetes, the way you’re acting and the way you are. And then his little sister is a year and a half younger, but she weighs more than he does. So, he’s still … he’s kind of frail, he’s thin, and you do notice that. We even had a period where we considered checking his blood sugar from time to time, because he just kept being unwell—tired, losing weight, pale—all those things that make me immediately think: could it be diabetes? So for us, this has put that worry on hold for a while. Now we can just look at him as a regular child and say, maybe you don’t have the strongest immune system, but there’s nothing else wrong.”* (Mom3 + diabetes, A).

The quote illustrates how, prior to participation in the early detection program, the family’s understanding of the child’s bodily signs was strongly shaped by a diabetes lens, in which ordinary symptoms such as tiredness, weight loss, or pallor were repeatedly interpreted as possible indicators of emerging disease. However, the quote shows how the early detection results temporarily disrupted this interpretative framework. Receiving a reassuring result allowed the parents to suspend this illness focused vigilance and to reframe the child’s symptoms as part of normal childhood variation rather than as early signs of diabetes. In this sense, early detection did not remove the underlying concern but introduced a provisional pause in the diabetes-oriented interpretation of symptoms, enabling the child to be seen—at least for a time—as a “regular child”.

A similar dynamic is articulated by another parent living with T1D, whose reflections highlight how personal illness experience intersects with the meaning ascribed to early detection results. While the underlying awareness of risk remains present, receiving numerical confirmation of a current low-risk status emerges as emotionally significant in managing uncertainty:

*“Yes, but I’ve always thought about it in relation to having it [T1D] myself. I know there’s some level of risk, so it’s something I’ve always reflected on. It’s really nice to get numbers that are at zero—or whatever they were—but at least show that they’re not currently in the danger zone. That’s very positive.”* (Dad1, + diabetes, A).

Even among parents who received a positive result indicating multiple autoantibodies and thereby a lifelong guarantee for the child to develop diabetes, the result itself did not necessarily appear to increase their level of concern significantly:

*“I’m actually somewhat relieved that the other two have tested negative. I can feel how com-forting that is—it’s one less worry. But I think once we get a few more results on [Son 3], and once we’ve had a good conversation with a doctor or someone from the research team who can explain the tests that were done and talk more about what the process looks like—then I think I’ll feel more at ease. It’s about knowing what comes next. Because that’s really what’s weighing on me—the uncertainty about what’s going to happen (…).”* (Mom4, -diabetes, A).

Mom4 expresses relief when learning that the two other children tested negative, describing this as comforting and as “one less worry.” However, her reaction to the result concerning Son 3 is characterized by continued unease rather than reassurance. She explains that she will only feel more at ease once additional results are available and once she has had the opportunity to speak with a doctor or a member of the research team who can explain the tests and clarify the next steps in the process. Thus, Mom4’s response to the result highlights that her primary concern lies not in the test result itself, but in the uncertainty surrounding “what comes next?”.

This reaction aligns with Sense of Coherence (SOC) theory, as the lack of comprehensibility and manageability regarding the subsequent process appears to undermine her sense of security. Consequently, clear information, predictability, and dialogue with healthcare professionals emerge as central to reducing uncertainty and supporting parents’ perceived sense of control throughout the early detection process.

In summary, Theme 1 illustrates how families’ experiences of early detection are shaped by preexisting diabetes narratives grounded in lived experience and emotional memory. Parents enter the early detection process with established worries, embodied knowledge, and well-developed interpretations of risk, which influence how test results and bodily signs are understood and emotionally processed.

While participation in early detection highlights emotional and psychological vulnerabilities related to uncertainty about disease progression and timing, it also reveals a high degree of parental reflection and familiarity with diabetes related risk. These families possess substantial experiential resources and disease specific knowledge that seems to enable them to engage proactively and meaningfully with the early detection process. This duality of concern and competence provides a foundation for Theme 2, which further explores how diabetes is embedded and pragmatically integrated into everyday family life.

### 5.2 Theme 2: Managing risk in everyday family life

Theme 2 examines how participation in early detection becomes embedded in families’ everyday understandings and practices related to diabetes. For parents who already live with diabetes in the family, screening is not experienced as an isolated medical procedure but as something interpreted through existing routines, experiential knowledge, and practical strategies for managing risk in daily life. The theme explores how families draw on experiential knowledge and pragmatic decision-making to integrate diabetes into daily life in ways that render it manageable rather than exceptional or crisis driven.

In contrast to the uncertainty expressed by Mom4 in Theme 1—where not knowing “what comes next” constituted a central source of distress—the following case illustrates how a clear understanding of the disease trajectory seems to activate substantial familial resources. During the following interview, this father makes little reference to the recent negative test result for his daughter; instead, he focuses on recounting the experience of recognizing the onset of diabetes in his son:

*“We discovered it on the way home from Jutland. One evening I had been watching a series with him, and I thought it was strange that we couldn’t get through a single episode of ‘Friends’ without him needing to go to the bathroom. Well, it could be many things. But on the drive home, we had to stop three times—and it’s a three-and-a-half-hour drive. Then we knew. We chose not to say anything that first day because we were going home to celebrate [daughter]’s birthday. That shouldn’t be ruined by sitting in a hospital (…).”*.

(Dad2, + diabetes, A).

Here, the father’s narrative reflects immediate recognition and interpretation of symptoms grounded in prior lived experience with T1D. Rather than generating anxiety or confusion, symptom recognition appears to bring clarity and direction. From a Sense of Coherence (SOC) perspective, this reflects strong comprehensibility, as bodily signs fit seamlessly into an existing illness narrative, and meaningfulness, as the situation is understood as serious but manageable and embedded in everyday family life.

When this family was referred to the pediatric department by their general practitioner a few days later, the parents, drawing on their extensive knowledge of diabetes, had managed to regulate their son’s blood sugar to such a degree, through exercise and a low-carbohydrate diet, that his blood glucose levels were within normal range. As a result, the healthcare professionals were initially skeptical about the family’s account of the boy’s condition:

*“(…) We ended up here [Herlev Hospital], where they said it wasn’t diabetes, but they put a sensor on him, and the levels were still fine—until we got so frustrated that we said, ‘Okay, you know what, go to 7 Eleven and get everything you want.’ And half an hour later, the staff came rushing out and said, yes, it is diabetes. [And we said] Well, we know that!”*.

(Dad2, + diabetes, A).

Encouraging the child to “go to 7 Eleven” functions as a deliberate and pragmatic strategy aimed at clarifying the situation and moving it forward. This response illustrates a strong sense of manageability, as the parents perceive themselves as having sufficient resources to influence the course of events and to engage assertively with professional authority. In contrast to the vulnerability described in Theme 1, uncertainty here is tempered by a strong sense of comprehensibility and manageability, allowing concern to coexist with calm, confidence, and action. What may initially appear paradoxical—that parents of first−degree relatives express high levels of worry while simultaneously mobilizing substantial resources—seems to reflect how uncertainty becomes less paralyzing when the situation is rendered understandable and actionable. When the course of the disease is perceived as known, uncertainty can transform into purposeful engagement rather than loss of control.

This sense of manageability also appears to shape families’ views on follow−up and continuity beyond the immediate screening encounter. Rather than perceiving early detection as a one−time event, some parents describe value in ongoing monitoring, which may further support a sense of predictability and reassurance over time. This perspective is exemplified by Mom3:

*“(…) It would be great if this was something that was offered from time to time. Given our situation, I think it would be really helpful to keep track of whether they might develop anything. Especially because [son] is quite fragile. I mean, he really … He’s been hospitalized more times than most of us will experience in a lifetime.”* (Mom3, + diabetes, A).

The quote illustrates that for these families participation in early detection does not necessarily lead to increased stress or anxiety, as this stress is already latent within the family. Early detection can on the opposite be experienced as a resource—a tool that restores a sense of agency and empowerment. It allows parents to actively engage with their child’s health trajectory, rather than passively awaiting a diagnosis. In this way, early detection is not only a clinical intervention, but also a form of psychosocial support in itself—providing families with emotional and practical preparation for the possibility of a chronic condition. Another mom further reflects on the significance of having access to annual early detection for T1D:

*“Well, it would give me tremendous peace of mind, because it’s my biggest worry. And it’s one of the reasons we had children so late—I simply didn’t want to pass my illness on. To be honest, I think it’s a horrible disease to live with* (…).

*“(…) It’s more important to be informed about something that might look concerning and be able to act on it, than to walk around with your eyes closed and just hope for the best. I’d rather receive a bad result and still be able to respond to it. When it comes to diabetes treatment, I believe the future lies in slowing the disease rather than curing it.”* (Mom5 + diabetes, A).

This mother’s belief in her own capacity to act reflects a high level of manageability, where she perceives that she has the resources and ability to respond to potential health threats. Mom5 also demonstrates how early detection gains meaning through hope for future treatment options, turning participation into a purposeful and forward-looking action rather than a source of worry. In some families, however, this hopeful orientation may delay reflection on potential consequences until results are received, as illustrated by Dad3:

*“I think you always hope for the best when you get an answer like that … The two of us haven’t really thought about, or talked about, what it would mean if one of the tests came back positive (…).”*.

(Dad3, + diabetes, W).

This illustrates how hope can function as a coping strategy that enables participation despite limited discussion of possible outcomes. While this reliance on hope may weaken comprehensibility and predictability, it nevertheless allows families to engage without becoming overwhelmed. At the same time, the data show that attention often centers on familiar conditions such as diabetes, whereas less familiar diseases may evoke greater anxiety due to limited knowledge and preparedness:

*“But I will say that there were three different things he could be screened for, and of those three, I was actually most concerned—if I had to say something—about the thyroid issue. I might be a bit more worried about that, because I honestly have no idea what that even is (…). I think that if [son] had tested positive for being at particular risk for type 1 diabetes, then I think we would’ve just gone into solution mode. Like, okay, let’s roll this out (…).”* (Mom5 + diabetes, A).

Like Dad1’s anecdote, the quote illustrates how knowledge about the condition fosters a sense of security and motivates families to take ownership and act. As this mother puts it, if her child were to test positive, “Okay, let’s roll this out” reflects a high degree of manageability and meaningfulness. While a T1D diagnosis is not desired, and the families hope for the best during the process, it does hold meaning for these families. The quote highlights the need for thorough communicative follow-up not only for diabetes, but also for the other conditions, which could provoke even more anxiety due to unfamiliarity.

The way communication between healthcare professionals/the research team and families are handled plays an essential role in the data, shaping parents’ level of concern and their ability to cope with the process—especially when receiving a positive result:

*“(…) What I find difficult right now is that I’m having trouble fully understanding the implications of the one antibody she tested positive for. I think the whole process has been strange. We had to call and follow up ourselves to get the results of the tests, and [daughter]’s results came with—I’m not entirely sure—about a six-week delay compared to [son]’s, even though their blood samples were taken within 30 seconds of each other.”* (Mom2 + diabetes, A).

Mom2 is frustrated over her lack of understanding regarding what it means that her daughter tested positive for one antibody. She describes the process as “strange,” suggesting that she experienced the early detection procedure as lacking meaningfulness and transparency. She further explains:

*“I just don’t get it—they took the tests 30 seconds apart, and the only explanation I got was, ‘We send them off once a month.’ And I’m like, okay, but if my kids had their samples taken 30 seconds apart, it would’ve been nice if you had informed me that you were sending them in two different batches, and that it could take that long (…) It just doesn’t make any sense. If I had brought them in on two different days, fine. But it was the same nurse, in the same room, at the same time—they didn’t even leave the room.”* (Mom2 + diabetes, A).

Mom2’s frustration illustrates how disruptions in transparency and delays in communication can rapidly reactivate the underlying uncertainty. This again suggests that prior knowledge not only facilitates coping but also heightens sensitivity to procedural ambiguities—an issue that was emphasized by several parents. In families where multiple children are tested simultaneously, delayed or staggered communication of results may therefore generate unnecessary worry, as parents are left to compare timelines and interpret asymmetries between siblings. Clear, timely, and consistent communication—including the delivery of results for siblings at the same time—plays a crucial role in sustaining parents’ sense of coherence throughout the early detection process, particularly when results are ambiguous or indicate increased risk.

Overall, the findings in Theme 2 show that families’ emotional and practical coping is shaped less by the presence of diabetes related risk itself than by how clearly the process is communicated and how well uncertainty is addressed by healthcare professionals. When parents are provided with timely information, transparent procedures, and a clear sense of direction, they appear able to draw on extensive experiential resources to manage risk in calm, pragmatic, and routine oriented ways.

These insights highlight where families of first-degree relatives feel most exposed and underscore the importance of early detection programs that support sense of coherence by strengthening comprehensibility and manageability alongside clinical surveillance. At the same time, findings from Theme 2 indicate that parents’ ways of coping with and navigating the early detection process may influence how participation is experienced within the entire family, including the children. Parents’ ways of handling information, uncertainty, and emotional responses may contribute to shaping the context in which early detection is introduced and discussed within the family. In light of this, theme 3 shifts focus to children’s involvement in early detection and explores how family practices may be reflected in children’s understanding of and responses to early detection.

### 5.3 Theme 3: Children’s involvement

Theme 3 examines children’s involvement in early detection as it unfolds within family practices of coping and meaning making. While the children were not interviewed directly, their perspectives emerge through parents’ descriptions of how they react to testing, risk information, and monitoring in everyday life.

In this theme, children’s age is included to enhance analytical transparency, as it provides context for how information is communicated and how uncertainty may be experienced at different stages of development. By drawing on parental accounts of children’s reactions and coping, Theme 3 extends the findings from the previous themes and shows how early detection is experienced within shared family dynamics, where parental ways of managing uncertainty constitute a central resource—and, at times, a point of vulnerability—for children.

Discussing the active involvement of children in the early detection process, the parents often described blood sampling as the most difficult part.

Mother of a 10-year-old girl and 7-year-old boy:

*“It was impossible to get the right amount of blood out of their finger, because it’s like a lot of blood that’s needed. And you must just squeeze this blood out. And for my son, or for both, I tried, it was so hard. And I got some blood out and there was blood everywhere, but it just wouldn’t go in the thing (…). So of course, the kids hated it (…).”* (Mom6 -diabetes, W).

Challenges related to blood sampling were particularly pronounced when using the home test kits, and to a lesser extent among families who had the opportunity to visit the diabetes center, where venous blood samples were taken by a professional. At the time of the interviews, blood sampling was only available at Steno Diabetes Center Copenhagen, and families living far from the center were therefor offered home test kits as an alternative. For some families, the blood test only amounted to a rough start to the process, while for others it meant they had to give up entirely, or that they only managed to get some of their children tested. However, the practical challenges with blood sampling, which many families encountered, were relatively minor compared to the more complex emotional difficulties that some families had to navigate during the process. Several parents reported that their children expressed concerns about developing diabetes, revealing the psychological weight of the process:

Father of an 8-year-old boy and 11-year-old girl:

*“Yes, and she [daughter] is generally quite worried. So you could say it has contributed to a concern like, “Oh, do I now have a chance of becoming diabetic?” Specifically, for her—my son is a bit more indifferent, he takes it more lightly—but when it comes to her, I’ve considered whether she should even participate in projects … I mean, going forward.”* (Dad5 + diabetes, A).

This quote shows that participation in the early detection program has intensified the daughter’s existing tendency to worry, particularly in relation to the possibility of developing diabetes. Rather than suggesting that she struggles to make sense of the information as such, the father’s account indicates that the notion of being “at risk” has become a source of concern for her. In contrast, her younger brother appears largely unaffected, highlighting how children within the same family may respond very differently to the same process. The quote also suggests that increased worry may lead parents to reconsider their children’s participation if the emotional costs outweigh the perceived benefits.

These differences underscore the importance of attending to children’s individual emotional responses when involving families in early detection, rather than assuming uniform reactions or needs for support.

Another father describes a different approach to communicating with his children about participation and risk:

Father of a 5-year-old girl and a 9-year-old girl:

*“Well, it’s been about being completely straight, like they can do a test, to see if they have diabetes. And then the oldest was kind of like, ‘Do I have it?’ [Dad’s answer]: I don’t think you do. I don’t know. But we can just test it, so that something preventive can be done, if it turns out that you do (…).”* (Dad6 + diabetes, W).

Taken together, the quotes illustrate variation in how parents talk to their children about early detection and risk, rather than demonstrating the effects of specific communication styles. In Dad6’s account, communication is described as open and concrete, while also acknowledging uncertainty. His explanation emphasizes testing and the possibility of preventive action, without offering firm reassurance or making definitive claims.

Dad6 further reflects on his own relationship with diabetes:

*“I’m pretty calm about it. And if it were to happen, it’s not the worst disease to have. It’s fine, I live almost normally. That said, I’m glad that I got it as an adult and not as a child (…).”* (Dad6 + diabetes, W).

Dad6’s reflection illustrates how parents’ own lived experiences with diabetes shape how risk and early detection are perceived and articulated within the family. From a Sense of Coherence (SOC) perspective, his calm and pragmatic stance reflects strong comprehensibility and manageability, as diabetes is framed as a known and livable condition. Empirically, this orientation appears to inform how he approaches early detection and discusses it with his children, contributing to a family context in which uncertainty is acknowledged but addressed with perspective and emotional steadiness.

In contrast, another parent with T1D explains why she chose not to share with her daughter a test result indicating multiple autoantibodies. Mother of a 7-year-old girl:

*“(…) So, she knows that she’s being tested. But we haven’t shared all the information with her. If she asks, she gets an answer. But she’s only seven years old, so if she doesn’t ask about it, it’s not … It doesn’t make a difference to her. She shouldn’t have to live and worry about something. But I actually don’t think she’s worried about getting diabetes either. Be-cause I have it. And she can see that I live a normal life. So I don’t think she’s worried about it.”* (Mom7 + diabetes, A).

On one hand mom7 wants to protect her child from having to live with this worry; on the other, she says she does not believe her daughter would be concerned if she knew—reflecting the thoughts and dilemmas this mother is considering in relation to sharing information with her daughter.

Another family actively declined the recommendation to return for follow-up blood testing within 6–12 months after receiving a positive result. The family’s youngest daughter had diabetes, and the two other daughters were therefore tested. The parents expressed concern that ongoing monitoring could unnecessarily increase their daughters’ anxiety without providing any immediate or actionable benefit. Mother of a 5-year-old girl, 12-year-old girl and 15-year-old girl:

*“I actually haven’t shared the result with her. Precisely because I didn’t want to tell one child, ‘You’re in the clear,’ while saying to [daughter 1], ‘You have a small risk.’ It was that same concern again—the worry I didn’t think she needed to carry around or have lingering in the back of her mind. If it ever becomes relevant, we’ll deal with it then.”* (Mom8 + diabetes, A).

Mom8’s account reflects a protective strategy aimed at shielding her daughter from worry and from being marked as “at risk.” At the same time, her decision not to share the result or to pursue follow-up testing illustrates a pattern identified earlier in the analysis: some parents enter the early detection process hoping for reassurance, while postponing engagement with what they would do if results indicates increased risk. In this sense, participation appears motivated by a desire to confirm that “everything is fine,” whereas positive or ambiguous results can challenge parents’ emotional readiness to proceed.

These concerns also reflect a broader ethical dilemma related to the risk of pathologizing children who may never develop T1D. The case illustrates the complex decisions families must navigate when balancing the potential benefits of early detection against the psychological burden associated with knowing and communicating risk. While the decision to withhold information is grounded in care and protection, it may also limit the child’s opportunity to gradually develop an understanding of her own health status.

The following quote from Mom4 illustrates how efforts to protect children from worry by limiting information may also have unintended consequences.

Mother of 3-year-old twins, a 6-year-old boy, and an 8-year-old boy.

*“You could see that he was very relieved when he was told his test was negative. Really relieved. He almost had tears in his eyes. (…) But that’s also because he reacted quite strongly when his brother was diagnosed with diabetes. And that might be part of what settled in his body and was then released by being told that there is a very, very low probability that he will ever get it.”* (Mom4 -diabetes, A).

This quote illustrates how receiving a negative test result can elicit a strong emotional response in a child, particularly when the family has prior experience with diabetes. Mom4 links her son’s relief to his earlier reaction to his brother’s diagnosis, suggesting that this experience has had a lasting emotional impact. The negative result appears to provide reassurance in the present situation and a sense of emotional release for this boy.

The quote illustrates how early detection results may activate or resolve emotions shaped by previous family experiences with illness. The material does not allow conclusions about how the child reacted earlier in the process, but it does indicate that test results can become emotionally meaningful moments where concerns—are brought to the surface. Across the material, parents reflected on how and when to involve their children in conversations about the early detection process, often expressing uncertainty about what information might be supportive versus potentially burdensome.

Mother of a 7-year-old boy and 10-year-old girl:

*“I really could have used some guidance on what is appropriate to tell my children. Should I share more about this with them, or would it have been enough just to have had some ad-vice? (…) Someone who isn’t my husband—who of course wants exactly the same for our children as I do—but rather someone who could help me reflect: What considerations should I make if I choose not to tell them everything?”* (Mom9 + diabetes, A).

Taken together, this underscores that early detection raises not only questions about testing and results, but also about parental responsibility and decision-making around what to share with the child and when. These reflections point to the importance of ongoing, attentive follow-up by healthcare professionals, particularly given that early detection provides only a provisional indication of a child’s health status. Despite prior experience with diabetes, parents frequently described early detection as unfamiliar territory, with communication with their children remaining a central and unresolved source of uncertainty.

While the preceding examples have focused on parents’ uncertainty and their need for guidance, attention now turns to children’s own coping strategies. The following example illustrates how an 11-year-old boy who tested positive for multiple autoantibodies appears to have developed his own ways of managing and making sense of the situation, including establishing a supportive space for reflection and dialogue outside the immediate family context. As his father describes:

Father of an 11-year-old boy:

*“He’s actually handled it quite well. One of his close friends in school also has type 1 diabetes, so they’ve talked about it for a long time. I haven’t noticed any changes in his behavior.”* (Dad7 + diabetes, A).

The quote suggests that the son has been able to place his experience within a meaningful context, which facilitates comprehension and emotional processing. The fact that he has talked about it ‘for a long time’ further indicates that he has had the opportunity to gradually process the information over an extended period. From a SOC perspective, this boy appears to perceive the situation as comprehensible rather than chaotic—he knows what it is about and has a frame of reference through his friend. His coping strategy contributes to making the upcoming ‘waiting period’ prior to a possible diagnosis feel significantly less stressful and will likely result in the waiting time being experienced as less burdensome.

Similarly, another 11-year-old boy responds to a positive result by making changes in his everyday life making the waiting period or the message about autoantibodies more manage-able for him.

Mother of an 11-year-old boy:

*“When the doctor called, we were in the car on our way to the summer house, and my son was listening in. He’s a very curious and perceptive child. He said, ‘Yes, Mom, he said it wasn’t much, but it could be something, though there’s nothing right now.’ We’re very open about these things. Of course, there are things you don’t always share with children, but in this case, we did—especially because we’ve had ongoing issues with him around eating and vegetables. He’s extremely picky. But that phone call actually made him reflect and think that maybe he should try to overcome his aversion, because it’s a huge challenge for him to try new textures. So for him, it led to a change—he started eating healthier.”* (Mom10 - diabetes, A).

This boy receives the message from the doctor together with his mom, and his summary of the doctor’s words shows that he understands the content and relates to it in a way that makes sense to him. He subsequently made active changes to his diet and started eating healthier, which might indicate that he feels he has options and resources to do what he can to try to improve the situation. From a SOC perspective, this child demonstrates a strong internal framework for coping. He understands what is happening, feels capable of acting, and engages meaningfully in his own health.

In summary, Theme 3 highlights that children’s involvement in early detection unfolds within broader family practices of coping and communication. Parents’ accounts show that children respond differently to testing, risk information, and monitoring, shaped by age, family experiences with diabetes, and how information is shared. Several parents also describe children developing their own coping strategies, for example by drawing on familiar reference points or making practical sense of the situation. At the same time, parents emphasize the importance of support from healthcare professionals in guiding what and how to communicate, so that children are sufficiently informed without being burdened by unnecessary worry.

## 6 Discussion

Our findings reveal a central paradox: while prior experience with type 1 diabetes constituted an important coping resource for families with first-degree relatives, it simultaneously increased emotional vulnerability. Consistent with the quantitative findings of Melin et al. (15), parents from first-degree relative families appeared more emotionally affected and anxious than parents from the background population. Our qualitative findings extend this knowledge by illustrating how and when such worry is activated. Unclear procedures, delayed communication, or insufficient follow-up readily reactivated parents’ embodied memories of disease onset and fears of progression. In this sense, early detection did not introduce entirely new distress, but temporarily intensified existing concerns rooted in lived experience. For these families, a negative test result was often described as a moment of emotional relief—an opportunity to pause and see their child as an ordinary, healthy child, rather than as potentially ill.

Interpreted through Antonovsky’s Sense of Coherence (SOC) framework, our findings suggest that early detection supports coping when it strengthens comprehensibility, through clear explanations and predictable timelines, and manageability, through access to healthcare professionals and explicit guidance on next steps (25). When these elements were present, families described early detection as something that could be pragmatically integrated into everyday life and expressed willingness to remain engaged in the process. When absent, uncertainty became emotionally destabilizing. This underscores that prior knowledge alone does not guarantee coping; rather, coping depends on how well early detection processes are communicated and supported.

Children’s involvement in early detection should similarly be understood as a supported and developmentally sensitive process. In line with the Lundy model of child participation (26), meaningful involvement requires that children are offered appropriate space to express their views, support to articulate them (voice), opportunities for these perspectives to be heard by healthcare professionals (audience), and that they are taken into account in follow-up and decision-making (influence). Our findings indicate that such involvement depends heavily on parental support and coherent professional guidance, rather than on expecting children to navigate risk and uncertainty independently.

Taken together, these findings point to a group of families with relatively strong coping capacities—yet these capacities cannot be assumed to be evenly distributed across the population. From an SOC perspective, coping resources are shaped by broader social and socioeconomic factors, such as education and social position. This suggests that families’ ability to manage early detection does not only shape their experiences within such programs but may also influence whether they engage in early detection at all. Families with fewer resources may perceive early detection as burdensome or anxiety-provoking and therefore opt out, raising concerns that early detection initiatives could unintentionally exacerbate existing health inequalities. This is particularly concerning given that families facing social disadvantage are also more likely to present with diabetic ketoacidosis at diagnosis and thus stand to benefit substantially from early intervention (27).

This concern aligns with evidence from other preventive health contexts, where participation bias has been well documented. Studies of cancer screening, for example, show that socioeconomic and demographic factors strongly predict non-participation (28). To promote equity in population-wide early detection of type 1 diabetes, integrating screening into existing universal child health services—such as routine health examinations or vaccination programs—could offer a more inclusive model. Evidence suggests that early detection between 2 and 6 years of age, with a possible repeat around age 10, is most effective (4, 16). Embedding early detection within established child health services, which achieve participation rates above 90% in Denmark (29), could reduce reliance on voluntary opt-in models and help mitigate socioeconomic bias. However, such an approach would require general practitioners to take on responsibility for follow-up, including providing clear explanations, emotional support, and appropriate education for families of children who test positive - representing a substantial demand on primary care.

The vulnerabilities identified among relatively resourceful families in this study may also be relevant when considering families with fewer resources. As early detection initiatives increasingly aim to reach broader and more diverse target groups, the following practice-oriented recommendations may therefore be of growing importance for the future screening of first-degree relatives:

Communication and Transparency.

- Provide clear and timely communication throughout the entire early detection process.
- Increase transparency through simple timelines or digital tools (e.g., sample tracking systems).
- Explain results in a structured manner, both verbally and in writing, and communicate results for siblings simultaneously.
- State next steps explicitly and communicate available support options, particularly for families receiving positive results (e.g., counseling, peer support programs, or helplines).

Supporting Children During Early Detection.

- Develop guidance for parents on how to support children emotionally and practically.
- Equip healthcare professionals with tools to coach and empower parents in these conversations.
- Offer age-appropriate education and emotional support for children at high risk or with multiple autoantibodies.

## 7 Strengths and limitations

One key strength of our study is that it, to the best of our knowledge, is one of the first qualitative investigations into the psychosocial dimensions of early detection in families of first-degree relatives. In doing so, it contributes novel and empirically grounded perspectives to a field in which qualitative evidence remains scarce. Furthermore, the study supports the broader development of early detection strategies in diabetes care, marking a shift toward more family-centered and preventive approaches. This is reflected in the practice-oriented recommendations, aimed to support high-risk families in navigating future early detection processes.

Another strength of the study lies in its rich qualitative dataset, comprising 31 interviews conducted with mothers, fathers, and in some cases both parents together. These interviews provided a nuanced understanding of how families navigate the early detection process and highlight specific areas where psychosocial support mechanisms could be enhanced.

One key strength is the use of the SOC framework to interpret parental experiences of T1D early detection. This provides a structured lens for understanding how parents make sense of, manage, and find meaning in the process, thereby deepening and nuancing the analysis of psychosocial dynamics. Structuring the findings around and within a theoretical framework also provides the opportunity to use the same framework when developing possible solutions and future interventions. Applying SOC strengthens the study’s grounding in existing psycho-social research and links its findings to broader strands of research on coping, communication, and family adaptation. This theoretical anchoring enhances analytical rigor and ensures that the findings will contribute meaningfully to existing evidence in pediatric healthcare.

Still, several potential limitations should be considered when interpreting our findings.

First, the participant sample may not fully reflect the broader population eligible for early detection. Some individuals may have overlooked the digital invitation or were not reached through other recruitment channels, while others may have actively declined participation. Consequently, perspectives related to non-participation—particularly motivations and concerns—are not captured in the present study.

Second, the study only includes interviews with parents, omitting the direct voices of the children. This limits our understanding of how children experience early detection and potential diagnosis, and for this reason, future research should aim to incorporate children’s perspectives directly.

Third, families from ethnic minority backgrounds are underrepresented in the sample. This is particularly concerning given that ethnic minorities seem to be at increased risk of diabetic ketoacidosis at diagnosis (30). This limitation requires attention moving forward, as failure to ensure that early detection initiatives reach families with an ethnic minority background could exacerbate existing health inequalities.

Finally, another limitation concerns the imbalance in the number of parents interviewed who themselves had diabetes compared to those without the condition. This skew was expected, however, given the overall composition of our participant sample.

## 8 Conclusion

This study set out to explore how families perceive and experience participation in an early detection program for type 1 diabetes. The findings show that participation is shaped not only by clinical procedures but by families’ lived experiences, emotional memory, and existing understandings of diabetes. Parents enter early detection with well-established narratives and experiential knowledge, which influence how risk information, bodily signs, and test results are interpreted and emotionally processed.

Across the analysis, families’ emotional, psychological, and practical challenges were closely linked to how early detection was communicated and supported. Parents described feeling particularly vulnerable in situations marked by unclear procedures, delayed communication, or unresolved uncertainty. Conversely, when information was timely, transparent, and accompanied by ongoing follow-up, families felt better supported in integrating early detection into everyday life in manageable and pragmatic ways.

The study further illustrates that parents’ experiences of participation shape how early detection is introduced and discussed within the family, including with children. Children’s responses varied depending on age, prior family experiences with diabetes, and how information was shared. While some children were unsettled by uncertainty, others developed their own ways of coping and making sense of risk in everyday contexts. Parents emphasized a need for healthcare support in navigating what and how to communicate, so that children are sufficiently informed without being burdened by unnecessary worry.

By focusing on parents’ lived experiences, this study highlights areas where families feel most exposed and in need of healthcare support when participating in early detection. The findings suggest that the implementation of early detection programs should attend not only to clinical accuracy but also to the emotional, relational, and ethical dimensions of participation. Designing and implementing programs that are responsive to families’ vulnerabilities, communication needs, and sense-making processes will be essential to ensuring that early detection remains clinically sound and meaningfully aligned with families’ needs in the future.

## Data Availability

The datasets presented in this article are not readily available because due to the highly sensitive and identifiable nature of qualitative interview data, the full dataset cannot be shared publicly. A description of the data collection and analysis procedures is provided in the article. Requests to access the datasets should be directed to pernille.bech.flarup@regionh.dk.
